# Studying the Same-Gender Preference as a Defining Feature of Cultural Contexts

**DOI:** 10.3389/fpsyg.2020.01863

**Published:** 2020-08-14

**Authors:** William M. Bukowski, Dawn DeLay

**Affiliations:** ^1^ Concordia University, Montréal, QC, Canada; ^2^ Arizona State University, Tempe, AZ, United States

**Keywords:** gender, culture, peer relations, same-gender preference, human development

## Abstract

Research on culture would be enriched by studying the connection between gender and peer relations. Cultures vary in the roles, privileges, opportunities, and right that are ascribed to girls and boys. They are known to also differ in the degree to which girls and boys interact with each other. Although the preference for same-gender peers has been observed across multiple cultural contexts, the degree of this segregation between girls and boys varies across contexts. We argue that variability in the divide between girls and boys is an important cultural feature of contexts that is likely to affect developmental processes and outcomes.

## Introduction

In this paper, we propose that a well-established finding from research on peer relations can be used to measure and understand diversity in gendered experiences across cultural contexts. The peer finding that interests us is the observation that, beginning in early childhood and continuing across the school-age years and into adolescence, girls and boys are more likely to associate with and like same-gender peers more than other-gender peers (Rubin et al., 2016). This divergence is meaningful in at least two ways. It provides insight into the degree to which gender functions as a social category that organizes interpersonal experiences. It also provides important descriptive information about the social structure of children’s peer-based developmental contexts. We argue that it can be used to expand our understanding of how gendered experiences within the peer group vary across cultural contexts and how these differences may affect development.

The same-gender preference is typically conceived of and measured at the level of the person. It refers to the degree to which a person prefers to like, befriend or become acquaintances with same-gender peers compared to other-gender peers. In this respect, it is perceived to be a form of personal preference. We propose that this same-gender preference can also be conceived as a feature of social groups. Our point is that this key component of gender segregation will vary in meaningful ways across contexts, and that these contextual variations have important consequences for basic forms of development. Finally, although we recognize the limitations of this decision, we have chosen to predominately reference traditional, binary gender categories (e.g., boy/girl and man/woman) in this text. This decision was made to allow for simplicity in the comparisons made across time and cultural contexts.

We recognize that culture can be a contested construct that is difficult to define (Geertz, 1995). We define culture as the activities and the related cognitions, attitudes, and values that are characteristic of a particular context (Ratner, 1999). The points we wish to make can be applied to a broad set of contexts. These contexts may be small, such as a classroom-based peer group or large, such as a nation state. They can also vary in their status as either formal institutional structures, such as a school context or informal voluntary groups, such as a fitness class at a neighborhood gym.

## The Same-Gender Preference

The same-gender preference has been widely replicated (Thorne, 1986; Maccoby and Jacklin, 1987; Maccoby, 1998). It has been observed across multiple studies using either observation-based measures of social interaction (Martin et al., 2013) or sociometric indicators of liking (Bukowski et al., 1993; Sippola et al., 1997; Burton-Smith et al., 2001; Poulin and Pedersen, 2007). Other studies of the same-gender preference have assessed differences in the degree to which children and adolescents expect to enjoy interacting with same- and other-gender peers (Strough and Covatto, 2002). Alternatively, some studies focus on the importance of respecting the boundary or the dividing line that keeps same- and other-gender peers apart from each other during preadolescence (Sroufe et al., 1993). Although research on the same-gender preference has been focused largely in the childhood and adolescent periods, there is evidence that it extends well into adulthood (Mehta and Strough, 2009).

The well-documented evidence that females and males of all ages tend to like and spend more time with their same-gender peers than with their other-gender peers is a traditional staple of the literature on both gender (e.g., Ruble et al., 2006) and peer relations (Rubin et al., 2016). It is one of the primary contact points between developmental research on gender and research on peer relations.

Based on the evidence of this gendered divergence in social experiences, Maccoby (1999) chose to describe the social contexts of girls and boys as “two cultures.” She used this term somewhat broadly to capture a general set of differences between what goes on in the peer interactions and relationships of girls and boys as well as the limited degree of contact between them. The generality in her use of the word “culture” presents strengths and weaknesses. Its strength is its capacity to summarize into a single term or concept the differences taken from different forms of functioning and from different levels of social complexity including the levels of the individual, dyad, and group. This breadth is also a limitation. As Underwood (2004) has noted, basing culture on a broad set of indicators taken from different forms of functioning prevents simple tests of the validity of the “two cultures” concept.

The study of the same-gender preference and of gender issues more generally, has received little attention in both the literature on peer relations and culture as well as on gender and culture. Gender rarely enters into culturally informed studies of peer relations. A similar comment can be made, albeit to a smaller degree and from a different perspective, about the study of gender and culture. Cultural analyses of gender have typically centered on issues of power, access to resources, privileges, and social roles. They highlight and reinforce the evidence that the different social roles, experiences, and opportunities that are ascribed to women and men vary across cultural contexts. Social and cultural analyses of gender have a group focus in the sense that women and men are perceived to constitute different groups within contexts. In spite of this apparent group focus, analyses often deal with social phenomena at the level of the individual. These comparisons typically examine the rights and personal experiences that are ascribed to individuals as a function of the gender category in which they are situated. In this way, their focus is on what individual people do, or can do, as a function of their gender rather than focusing on gender as a group level construct or category.

This emphasis on the rights, roles, and privileges that are ascribed to individuals as a function of their gender, fails to capture differences in the degree to which individuals see the same-gender and the other-gender as distinct social groups—or the degree to which the same-gender group is taken to be the primary domain of social participation. It is known already that, in some contexts, strong prescriptions keep men and women apart, especially in the social sphere. This divergence between women and men, or between the same and the other, can be the result of well-established traditions reinforced by institutional practices. As an example, in some groups, men and women are not allowed to touch each other, except in narrowly defined personal circumstances (Feldheim, 2013); girls and boys cannot be students in the same primary and secondary school classrooms (Bahrami et al., 2016); and in some houses of worship, separate sections are designated for male and female members of the congregation (Sorin, 2001). In contrast, in other places, individuals are allowed to associate more freely with members of the other gender. They can work and socialize together without formal concerns about crossing a boundary that keeps the same and the other apart. Nevertheless, behavioral practices are assumed to coexist in these contexts as well, such that members of the other gender are truly seen to be the “other.” Thus, even in contexts where gender integration is not proscribed by particular rules or norms, it can nevertheless occur. It should be noted that network analyses of social groups in different cultural contexts consistently reveal a same-gender bias in social selection processes (for reviews see Martin et al., 2013; Veenstra et al., 2013).

## Context, Gender, and the Salience of the “Other”

The concept of the “other” has been a main stay of theory and research on gender conducted by developmental psychologists. Starting in early childhood, children can identify the gender group to which they belong and have a set of schemas about gender that guide them to approach or avoid behaviors that are “appropriate” or “inappropriate” for their gender (Martin and Halverson, 1981; Martin, 2000; Ruble et al., 2006). Children’s understandings of gender are believed to create an in-group orientation that guides them toward gender-typed activities and toward same-gender peers and away from other-gender peers (Maccoby, 1998; Martin and Fabes, 2001). The concept of the “other” is especially prominent in developmental intergroup theory (Bigler et al., 1997). It claims that the salience of social group memberships is the result of context-based practices that clearly delineate social category memberships, including gender. These concepts are typically used to explain, in part, their preference for same-gender peers.

A direct consequence of category salience is the infrequent levels of interaction between individuals from different groups. Other consequences that come from this infrequent interaction may be more insidious and have stronger developmental effects. One of these effects may be the limited level of exposure that children and adolescents have to the norms, expectations, and practices of the other-gender group. This restricted exposure to a broader set of standards and beliefs has the potential to create a narrow perspective on how to function with other-gender peers and on available opportunities for self-presentations and self-perceptions. These processes may create a very constrained and canalized sense of what it means to be a female or male or even what it means to be a “normal” person. Another consequence may be a reinforcement and reification of the legitimacy of well-defined gender categories. The creation of inflexible gender conceptions is likely to have especially negative costs for children and adolescents who do not see themselves as fitting into traditional gender categories.

The issue of contextual variance in category salience that is central to intergroup theory is also seen in one anthropologist’s reflections on the origins of some cultural dimensions. Clifford Geertz (1995) pointed to this topic in his descriptions of the language training he received in preparation to work on field projects in Morocco and Indonesia. He explained that the person teaching him Arabic would admonish him harshly when he made gender-based grammar errors, whereas the person teaching him the Javanese language was more concerned with mistakes with status-related terms. Geertz demonstrates that while place/language gender was a critical form of social distinction in one place, status was a salient factor in another. Geertz’s point reinforces the central claims of intergroup theory, specifically that the importance ascribed to a social category is contextually variant and that membership in these categories leads to conformity to the group’s norms for some forms of social participation.

This convergence of ideas across scientific disciplines (e.g., developmental psychology and anthropology) and the widespread recognition that the salience of social concepts varies across social/cultural contexts point to potentially using the same-gender preference as a means to better understand variations between social contexts. Developmental psychologists have used the same-gender preference as a way of describing the features of the peer group and of assessing how the peer system changes with age. Studies of variations in the same-gender preference have typically focused on differences between individual children using measures of gender schemas (Powlishta et al., 1993), social behavior (Sippola et al., 1997), and activity preferences (Martin et al., 2013) all measured at the level of the person. Contextual analyses of variations in the same-gender preference have been far less frequent. Given that a basic point of intergroup theory is that the salience of gender categories will derive from environmental/contextual factors, understanding between-context differences would appear to be of critical importance. This general inattention to place differences is not entirely surprising in light of the overall paucity of research on cultural differences in many aspects of development.

## Context Differences in the Same-Gender Preference

An important exception to the lack of attention paid to contextual differences in the same-gender preference is the study of Whiting and Edwards (1988) of the social interactions between girls and boys in different contexts including communities in India, Kenya, Mexico, Peru, Liberia, Guatemala, the Philippines, Okinawa, and the United States. In this extensive study, titled *Children of Different Worlds*, Whiting and Edwards made careful observations of the amount of contact that girls and boys had with their same-gender and other-gender peers. From the outset, they used concepts from theory and research on cognitive development to form hypotheses about age differences in the preference for same-gender peers. Similar to other researchers (e.g., Maccoby, 1999), they expected that the rigid use of categories by young children would promote a strong in-group identification that would lead to a same-gender preference. They also proposed that a basic curiosity to know what it means to be a male or female within their own culture would lead children to pay more attention to the peers whom they perceived to be like the self rather than to those whom they perceived to be different. In this way, they ascribe a functional self-related purpose to having a primary affiliation with same-gender peers. It is important to note that an implicit feature of this reasoning is that gender role conformity is a consequence of gender segregation rather than an antecedent of it.

The study of Whiting and Edwards (1988) of *Different Worlds* revealed similarities and variability across the contexts they studied. A general preference for same-gender peers was observed in each of the contexts they observed. In every community, school-age children were more likely to associate with members of their own gender than members of the other gender. More importantly, the magnitude of this preference varied considerably across contexts. For example, when children’s peer interactions were observed in a free-play context, children in all communities were more likely to be in gender segregated peer groups. In one Kenyan community, the percentage of children in same-gender peer groups was 55, collapsed across boys and girls. The corresponding percentages for children from two other communities in Kenya as well as in communities in Guatemala, the United States, and Peru were 63, 74, 61, 80, and 100%. The variance across these groups is, to us, vastly more impressive than the claim that groups in each community show a same-gender preference.

Strough and Cavatto (2002) have noted that in Gold and Gold’s (1982) comparative study of school-age children in Sweden, Australia, England, and North America, the same-gender preference was weaker with the Swedish children than for the children from the other contexts. A more complicated set of findings observed by Cohen et al. (1980) points to the challenge of assessing the degree of desired contact with the other gender. In their study, preadolescent children from Sweden and the United States were asked to choose the peers whom they would like to have as a partner in a school-related task (i.e., help with homework) or in a personal exchange (i.e., sharing a secret). Three important findings were observed. First, on the school task, 34% of the boys from the United States chose at least one other-gender peer, whereas only 5.3% of the girls did the same. No differences were observed between the Swedish girls (11%) and boys (12%). Second, similar findings were observed with the personal task. The boys from the Unites States chose an other-gender peer more frequently (18%) than did the girls from the United States (2%). Again, no differences were observed between the Swedish girls (19%) and boys (14%). Third, a direct comparison demonstrated that the American boys (34%) were more likely than the Swedish boys (12%) to choose a girl for the school task, but there were no differences on the personal task (18 and 14%, respectively). A different pattern was observed with the girls. On both tasks, boys were chosen more frequently by Swedish girls than by girls from the United States (11.4 and 5.3% for the instrumental task and 18.6 and 1.8% for the expressive task for the American and Swedish girls, respectively). These findings indicate that the tendency to choose other-gender peers as associates varies as a function of context, type of task, and gender.

The findings of contextual variations reported by Whiting and Edwards (1988) and by Cohen et al. (1980) are intriguing. They reveal substantial between-context variability in the orientation toward same- and other-gender peers. It is hard to overlook the variability revealed by their findings. At the same time, however, this is only a beginning as these data are descriptive rather than explanatory. They present evidence of variability but they fail to explain the source or the consequences of this variability across national contexts.

## Same-Gender Preference as Experience

The same-gender preference is, of course, much more than just an interesting descriptive feature of children’s and adolescent’s peer groups. Instead, it is a powerful structural factor that defines the social-developmental environment across the lifespan, from childhood through adolescence and into adulthood. By affecting how and with whom a child can, or should, associate, these social and cultural barriers and/or biases have a direct effect on the day-to-day experiences and interactions that children and adolescents have in social spaces, particularly in the school context. Moreover, by providing direct evidence of the dimensions that organize the social context, these same-gender preferences may reinforce the constructs that account for intergroup conditions. Such exclusionary preferences may also reify and strengthen the belief in rigid and traditional gender categories.

Contextual variations in the strength of the same-gender preference are also likely to have important consequences on development. Peer experiences, for example, are known to promote well-being *via* several processes including opportunities for acceptance and validation, the promotion of social skills, and protective experiences that minimize the effects that may result from negative experiences within the family (Rubin et al., 2016). In this capacity, interactions and relationships with peers can function as social assets and developmental protective factors. When access to other-gender peers is foreclosed by a sharp divide between the same- and the other-gender peer groups, the range of these beneficial functions of peer relationships can be limited.

Two processes may be especially important to consider. The first is that segregation by gender limits access to social capital. It is known that well-being derives from being accepted by both same- and other-gender peers (Bukowski et al., 2017). Seclusion to one’s own gender minimizes access to the benefits of being accepted by the other gender. The second process is concerned with protective factors. Some children are not liked by same-gender peers (Bukowski et al., 1999). Within the same-gender peer group, they may be friendless or have very few positive social connections or many negative social connections as a function of peer rejection and bullying. For these children, positive and supportive other-gender peer relationships may provide an important protective refuge. Thus, for such children, opportunities for interactions and relationships with other-gender peers may have the potential to reduce the negative effects of problems with same-gender peers. When these opportunities are not available because of a structural and/or socially normative separation between the two gendered peer groups, the possibility of finding a protective shelter with other-gender peers may be limited.

Between-context differences in the orientation toward the same- and other-gender peers are also likely to have consequences for gender identity development. Gender identity development is a complex and multifaceted construct (Perry et al., 2019). We speculate that two of its basic components may be affected by variations in gender segregation. One of these components is the degree to which a person identifies with being a member of their own gender category (i.e., being a female or male). It is likely that this aspect of gender identity will be stronger in contexts, where gender segregation is strong. It is also the case that the negative consequences of not identifying with one’s gender category may be more drastic in highly gendered contexts. A second aspect of gender identity that may vary as a function of the same-gender preference may be the level of flexibility in the features that define what it means to be female or male. One can predict that greater flexibility will be observed when gender segregation is weak. That is, one can expect that gender roles will be more fluid or less fixed when there is a more balanced orientation toward same- and other-gender peers.

This balance may be especially important in the current historical context. The discursive landscape about characteristics linked to sex and gender and the intersection between them has never been as open and as active as it is in the present moment. Ideas about what it means to be a woman or a man and how the concepts of femininity and masculinity are defined and expressed have been issues throughout history. In the present moment, however, these issues have become especially poignant. More so than at any other time, discussions of the variability in the features that define the biological sex-based categories of women and men and that define the social gender-based categories of female and male appear on a nearly daily basis in multiple forms and media. This attention has been accompanied by a movement away from a binary conceptualization of gender categories toward more fluid and non-binary definitions and labels.

A full assessment of the claims we have made about the importance of assessing the gender segregation as a feature of cultural contexts may need to be studied from a multilevel approach. The consequences of being in a small context, such as a school environment, in which there is a high level of gender segregation may vary as a function of the prevailing level of segregation in the broader social environment, such as the community where the school is located. The effects of gender segregation may also need to consider the degree to which it promotes or limits cross-gender comparison. It has been reported that within-gender social comparisons decrease gender differences in self-representations, whereas between-gender comparison increases gender differences (Guimond et al., 2006). If, as we have proposed, high levels of gender segregation foster a view of the other gender as the “other,” then one can expect individuals in these contexts to see themselves in more stereotypical manners. Alternatively, it may be that gender segregation will have the opposite effect. It may promote within-gender comparisons that, in turn, will decrease gender differences in self-representations. Along these same lines, gender segregation may be motivated by multiple factors, including religious traditions, historical factors, and culture-based conceptions of gender. The reasons underlying gender segregation may moderate the effects of functioning in a context with a division between males and females. Accordingly, a key feature of research on the effects of gender segregation should be an assessment of whether the strength and features of these effects vary as a function of the cultural factors that cause this divide. All of these issues need to be addressed in empirical studies.

## Conclusion/Bottom Line

Our main point is that there is value in studying gender, peer relations, and culture in an integrated manner. An initial starting point for a research program that brings these domains together should be variations in the same-gender preference. This construct provides a direct means of characterizing societies according to the affective and participatory divide between females and males. It is a feature of the day-to-day experiences of females and males across the lifespan. Already, there is evidence that the same-gender preference varies across cultures. This evidence that the cultural variation in a gender-related construct is manifested in a form of social experience is consistent with the claim of Geertz (1973) that “Behavior must be attended to…because it is through the flow of behavior – or, more precisely, social action – that (cultural) forms find articulation” (p. 17).

There are practical implications of this work as well. To consider these implications, let us first review what we know. First, we know that gender segregation occurs. Second, we know that gender segregation occurs across the lifespan, within various groups, structural settings, nation states, and cultures. We importantly realize, however, that there are also significant and potentially meaningful variations in the degree of gender segregation across contexts. Thus, if we aim to move toward a more gender integrated – rather than segregated – world, we must first recognize baseline similarities (i.e., gender segregation is likely to occur) as well as differences (i.e., gender segregation will vary across contexts). Accounting for these similarities and differences in our research programs will help us to also more accurately anticipate the degree, source, and outcomes associated with gender segregation within and between various contexts. It is only through such an adaptive understanding that we can begin to think about designing intervention and prevention efforts to support a more gender integrated and socially equitable world.

A key advantage of studying of gender segregation is that it can be studied with simple, easy-to-implement techniques. Many studies of the same-gender preference have used traditional sociometric methods to measure the degree to which children are drawn to same- and other-gender peers. These data can be used to assess the same-gender preference at the level of each individual child; these data can then be used to create group means. These same data can be used in network analyses to create indices of gender integration. Regardless of how one analyzes the data, the study of cultural variations in the same-gender preference provides a powerful and direct way of learning about gender, peer relations, and culture all within the same analysis.

## Author Contributions

All authors listed have made a substantial, direct and intellectual contribution to the work, and approved it for publication.

### Conflict of Interest

The authors declare that the research was conducted in the absence of any commercial or financial relationships that could be construed as a potential conflict of interest.

## References

[ref1] Bahrami N. Sibmar M. Bukowski W. M. Vedadhir A. Panarello B. (2016). Factors that promote and impede other-sex friendships: a qualitative study of Iranian adolescent girls. Int. J. Adolesc. Med. Health 30. 10.1515/ijamh-2016-0067 27768583

[ref2] Bigler R. S. Jones L. C. Lobliner D. B. (1997). Social categorization and the formation of intergroup attitudes in children. Child Dev. 68, 530–543. 10.2307/1131676, PMID: 9249964

[ref3] Bukowski W. M. Gauze C. Hoza B. Newcomb A. F. (1993). Differences and consistency in relations with same-sex and other-sex peers during early adolescence. Dev. Psychol. 29, 255–263. 10.1037/0012-1649.29.2.255

[ref4] Bukowski W. M. Panarello B. Santo J. B. (2017). Androgyny in liking and in being liked are antecedent to well-being in pre-adolescent boys and girls. Sex Roles 76, 719–730. 10.1007/s11199-016-0638-6

[ref5] Bukowski W. M. Sippola L. K. Hoza B. (1999). Same and other: interdependency between participation in same-and other-sex friendships. J. Youth Adolesc. 28, 439–459. 10.1023/A:1021664923911

[ref6] Burton-Smith R. Davidson J. Ball P. (2001). Age-related variations and sex differences in gender cleavage during middle childhood. Pers. Relat. 8, 153–165. 10.1111/j.1475-6811.2001.tb00033.x

[ref35] Cohen J. J. D’Heurle A. Widmark‐Petersson V. (1980). Cross‐sex friendship in children: Gender patterns and cultural perspectives. Psychology in the Schools 17, 523–529.

[ref7] Feldheim M. (2013). Balancing women’s rights and religious rights: the issue of bus segregation. Shofar 31, 73–94. 10.1353/sho.2013.0013

[ref8] Geertz C. (1973). The interpretation of cultures. New York: Basic books.

[ref9] Geertz C. (1995). After the fact: Two countries, four decades, one anthropologist. Cambridge, MA: Harvard University Press.

[ref10] Gold R. Gold J. (1982). Children’s sexual thinking: a comparative study of children aged 5 to 15 years in Australia, North America, Britain, and Sweden. London: Routledge and Kegan Paul.

[ref11] Guimond S. Chatard A. Martinot D. Crisp R. J. Redersdorff S. (2006). Social comparison, self-stereotyping, and gender differences in self-construals. J. Pers. Soc. Psychol. 90, 221–242. 10.1037/0022-3514.90.2.221, PMID: 16536648

[ref12] Maccoby E. E. (1998). Gender segregation: childhood, adolescence, and adulthood. Gender and relationships: a developmental account. Questions of gender Perspectives and paradoxes 294–305.

[ref34] Maccoby E. E. (1999). The two sexes: Growing up apart, coming together. Vol. 4 (Harvard University Press).

[ref14] Maccoby E. E. Jacklin C. N. (1987). “Gender segregation in childhood” in Advances in child development and behavior. Vol. 20 ed. Reese H. W. (New York: Academic Press), 239–287. 10.1016/s0065-2407(08)60404-8 3630812

[ref15] Martin C. L. (2000). “Cognitive theories of gender development” in The developmental social psychology of gender. eds. Eckes T. Trautner H. M. (Mahwah, NJ: Lawrence Erlbaum Associates), 91–122.

[ref16] Martin C. L. Fabes R. A. (2001). The stability and consequences of young children’s same-sex peer interactions. Dev. Psychol. 37, 431–446. 10.1037/0012-1649.37.3.431, PMID: 11370917

[ref17] Martin C. L. Halverson C. F. Jr. (1981). A schematic processing model of sex typing and stereotyping in children. Child Dev. 52, 1119–1134. 10.2307/1129498

[ref18] Martin C. L. Kornienko O. Schaefer D. R. Hanish L. D. Fabes R. A. Goble P. (2013). The role of sex of peers and gender-typed activities in young children’s peer affiliative networks: a longitudinal analysis of selection and influence. Child Dev. 84, 921–937. 10.1111/cdev.12032, PMID: 23252713 PMC3628293

[ref19] Mehta C. M. Strough J. (2009). Sex segregation in friendships and normative contexts across the life span. Dev. Rev. 29, 201–220. 10.1016/j.dr.2009.06.001

[ref20] Perry D. G. Pauletti R. E. Cooper P. J. (2019). Gender identity in childhood: a review of the literature. Int. J. Behav. Dev. 43, 289–304. 10.1177/0165025418811129

[ref21] Poulin F. Pedersen S. (2007). Developmental changes in gender composition of friendship networks in adolescent girls and boys. Dev. Psychol. 43, 1484–1496. 10.1037/0012-1649.43.6.1484, PMID: 18020826

[ref22] Powlishta K. K. Serbin L. A. Moller L. C. (1993). The stability of individual differences in gender typing: implications for understanding gender segregation. Sex Roles 29, 723–737. 10.1007/bf00289214

[ref23] Ratner C. (1999). Three approaches to cultural psychology: a critique. Cult. Dyn. 11, 7–31. 10.1177/092137409901100102

[ref24] Rubin K. H. Bukowski W. M. Bowker J. (2016). Children in groups. In The handbook of child psychology. Lerner R. (Series ed.) and Bornstein M. (Volume ed.). 7th Edn, New York: Wiley, 175–222.

[ref25] Ruble D. N. Martin C. L. Berenbaum S. A. (2006). Handbook of child psychology. Gender development. 6th Edn. Vol. 3 Hoboken, NJ: Wiley and Sons, 858–932.

[ref26] Sippola L. K. Bukowski W. M. Noll R. B. (1997). Dimensions of liking and disliking underlying the same-sex preference in childhood and early adolescence. Merrill-Palmer Q. 43, 591–609.

[ref27] Sorin G. (2001). Beyond the synagogue gallery: finding a place for women in american judaism. J. Am. Hist. 88, 664–665. 10.2307/2675167

[ref28] Sroufe L. A. Bennett C. Englund M. Urban J. Shulman S. (1993). The significance of gender boundaries in preadolescence: contemporary correlates and antecedents of boundary violation and maintenance. Child Dev. 64, 455–466. 10.2307/1131262, PMID: 8477628

[ref29] Strough J. Covatto A. M. (2002). Context and age differences in same-and other-gender peer preferences. Soc. Dev. 11, 346–361. 10.1111/1467-9507.00204

[ref30] Thorne B. (1986). “Girls and boys together but mostly apart: gender arrangements in elementary schools” in Relationships and development. eds. Hartup W. W. Rubin Z. (New Brunswick, NJ: Rutgers University Press), 89–109.

[ref31] Underwood M. K. (2004). Gender and peer relations: are the two gender cultures really all that different? In Children’s peer relations: From development to intervention. Decade of behavior. eds. Kupersmidt J. B. Dodge K. A. (Washington, DC, US: American Psychological Association), 21–36.

[ref32] Veenstra R. Dijkstra J. K. Steglich C. Van Zalk M. H. (2013). Network–behavior dynamics. J. Res. Adolesc. 23, 399–412. 10.1111/jora.12070

[ref33] Whiting B. B. Edwards C. P. (1988). Children of different worlds: The formation of social behavior. Harvard University Press.

